# Knowledge, experiences, and practices of women affected by female genital schistosomiasis in rural Madagascar: A qualitative study on disease perception, health impairment and social impact

**DOI:** 10.1371/journal.pntd.0010901

**Published:** 2022-11-07

**Authors:** Angela Schuster, Bodo Sahondra Randrianasolo, Oliva Onintsoa Rabozakandraina, Charles Emile Ramarokoto, Dorthe Brønnum, Hermann Feldmeier

**Affiliations:** 1 Charité –Universitätsmedizin Berlin, corporate member of Freie Universität Berlin, Humboldt-Universität zu Berlin and Berlin Institute of Health, Institute of General Practice, Berlin, Germany; 2 Association K’OLO VANONA, Antananarivo, Madagascar; 3 Centre for Clinical Research, North Denmark Regional Hospital, Hjoerring, Denmark; 4 Charité –Universitätsmedizin Berlin, corporate member of Freie Universität Berlin, Humboldt-Universität zu Berlin and Berlin Institute of Health, Institute of Microbiology, Infectious Diseases and Immunology, Berlin, Germany; Federal University of Agriculture Abeokuta, NIGERIA

## Abstract

**Background:**

Female genital schistosomiasis (FGS) is a neglected manifestation of urogenital schistosomiasis caused by *S*. *haematobium*. The disease presents with symptoms such as pelvic pain, vaginal discharge and bleeding and menstruation disorders, and might lead to infertility and pregnancy complications. The perspectives of women with FGS have not been studied systematically. The aim of the study was to understand knowledge, experiences, and practices of women with FGS.

**Methods:**

We performed a qualitative study with seventy-six women diagnosed of having FGS, in the Ambanja district in Northwest Madagascar. Data collection was either through focus group discussion (N = 60) or in an individual semi-structured interview (N = 16). FGS was diagnosed by colposcopy. The data was analysed using Mayring´s qualitative content analysis.

**Results:**

Knowledge on how the disease is acquired varied and ideas on prevention remained vague. Patients suffered from vaginal discharge and pelvic complaints. Some women expressed unbearable pain during sexual intercourse and compared their pain to an open wound being touched. FGS considerably impaired women´s daily activities and their quality of life. Infertility led to resignation and despair, conflicts with the partner and to social exclusion from the community. Women fearing to sexually transmit FGS refrained from partnership and sexual relations. Many women with FGS reported stigmatisation. A coping strategy was to share strain with other women having similar complaints. However, concealing FGS was a common behaviour which led to social isolation and delayed health care seeking.

**Conclusions:**

Our study underlines that FGS has an important impact on the sexual health of women and on their social life in the community. Our results highlight the importance of providing adequate health education and structural interventions, such as the supply of water and the provision of sanitation measures. Further, correct diagnosis and treatment of FGS in adolescent girls and women should be available in all *S*. *haematobium*-endemic areas.

**Trial registration:**

The qualitative study was embedded in a randomised controlled trial (RCT) in which two doses of praziquantel were compared (https://clinicaltrials.gov/ct2/show/NCT04115072).

## Author summary

Female genital schistosomiasis is a parasitic disease, acquired by humans when exposed to infested water. Patients can develop severe gynaecologic symptoms and face psychological and social problems. Although urogenital schistosomiasis is very common in many communities in Sub-Sahara Africa, knowledge on FGS among community members and health care workers is poor. Knowledge and experiences of women affected by FGS have never been explored but need to be understood to provide effective health care and to promote adequate interventions.

In this study, waterborne and sexual transmission were the most recurrently mentioned pathways for FGS acquisition. Most women perceived the risk for FGS as immutable and related to the precarious living conditions. The women were able to differentiate between urinary and female genital schistosomiasis. FGS caused partnership conflicts and affected women’s position in the community. Stigma within the community and mistrust in health care resulted in delayed seeking health care. In view of these results, we propose including health education and structural interventions to improve living conditions, in order to reduce the burden of FGS. Further, improved access to correct diagnosis and treatment should be provided.

## 1. Introduction

FGS is considered as the most important gynaecological health threat in countries where urogenital schistosomiasis is endemic [1,2]; being both common and causing severe symptoms and complications: Community-based studies indicated that FGS is highly prevalent in sub-Saharan Africa including Madagascar [3–9].Prevalence among women varied between 15% and 75% depending on the setting and the accuracy of the diagnostic method used [3–9]. In Madagascar, prevalences between 15% and 52% have been reported [5,10,11,12]. FGS may already be prevalent in prepubertal and adolescent girls [8,13].

FGS can affect the ovaries, fallopian tubes, uterus, cervix, vagina, clitoris and vulva [14–16]. Inflammation occurs in all layers of the genital tissue, including the small blood vessels [17,18]. Internal and external genital organs may be affected simultaneously or subsequently [9,19,20].

Resulting from this pathomechanism, women with FGS experience genital pain, particularly during sexual intercourse, contact bleeding, vaginal discharge and menstruation irregularities [8,9,16]. FGS is associated with poor pregnancy outcome such as stillbirth, spontaneous abortion, and ectopic pregnancy [20]. Studies indicate that FGS causes primary or secondary infertility [15,21,22].

Although the medical and socio-cultural impact of FGS is considerable, health care providers and the scientific community alike have neglected the disease [3,6].

In 1978, the WHO Expert Committee on Epidemiology and Control of Schistosomiasis identified the importance of social and economic hurdles for the control of schistosomiasis. The expert committee underlined that “comprehensive understanding of environmental, demographic, societal, human behavioural and economic factors in schistosomiasis is essential for the design of control programs that are successful in the long run”[23]. Women and girls are at great risk to acquire schistosomiasis due to gender roles and traditional distribution of household chores [24]. The social role of women in paternalistic societies impairs their access to health facilities. Besides, fear of stigmatisation might hinder women to disclose FGS-associated symptoms [10].

Studies on FGS in Ghana and Tanzania have pointed out that knowledge about FGS in the general population and among health workers is low and misconceptions are common [25,26]. Hitherto, knowledge, experiences and practices of women with FGS have received little or no attention. Thus, we formulate the following research question: *“What do women with FGS in Madagascar know about the disease and what are their experiences related to FGS*?*”* By the time of submission, there were no other studies that had investigated knowledge, experiences and practices in FGS affected women in Madagascar.

## 2. Methods

### 2.1 Ethics statement

The study was approved by the National Committee of Ethics of Madagascar (Authorisation N°: 098-MSANP/CERBM; N°:059-MSANP/CERBM; N°:065 MSANP/SG/-AGMED/CNPV/CERBM). In line with Good Clinical Practice, the goals of the study were explained to each participant of the SSI or FGD in plain words in Sakalava or in simple Malagasy before enrolment. The right to withdraw was explained and enough time was given to discuss doubts and open questions. Each participant signed the informed written consent form and received a copy of the form. Since adolescent girls can be affected by FGS from puberty, and life-threatening sequels of FGS may already occur in this age group, we considered it unethical to exclude 15-17-years old patients from the qualitative study. To respect confidentiality of the adolescent participants, the informed consent of a third party (parent or legal guardian) was not asked for, as recommended by the Ethics Committee.

### 2.2 Study area and population

Madagascar is the fourth largest island in the world and has 25 million inhabitants, 60% of whom are under the age of twenty-five [27].The fertility rate is 440 per 100,000 live births: thus Madagascar is one of the most rapidly growing populations worldwide [28]. Seventy-five percent of the inhabitants of Madagascar live on less than 1.9 US Dollar a day, and most households have no access to electricity [29]. Christian missionaries introduced current social norms during the colonial period (1894 to 1960). For a long time, monogamy and conservative sexuality of women were the mainstay of Christian religion [30].Today, few couples practice Christian marriage, and cohabitation and premarital sexual relations are common [31].

The study was carried out in the Ambanja district in Northwest Madagascar, an area highly endemic for *S*. *haematobium*. The population is predominantly rural and derives its income from the cultivation of rice and cocoa. Some farmers also produce spices (vanilla, pepper, pink berry), or raise perfume plants (ylang-ylang, vetiver, patchouli). Income from cash crops is supplemented by fishing. Many women work in paddy fields along riverbeds and ponds. Water for personal hygiene, human consumption and animals is fetched from ponds, lakes, and small rivers. Open urination and defecation are common.

**Fig 1:**
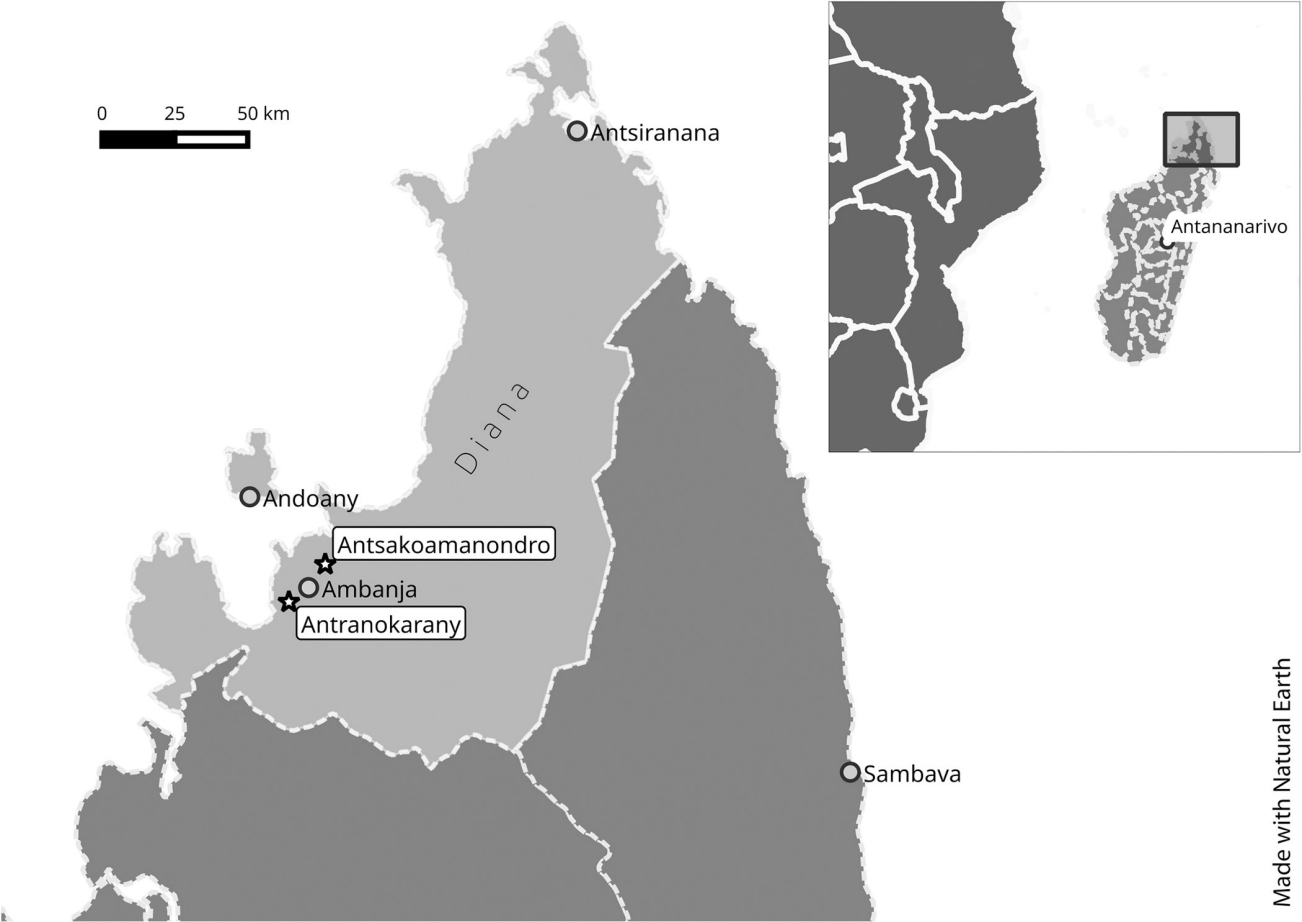
Map of the study area, made with Natural Earth (www.naturalearthdata.com).

The Sakalava represent the main ethnic group in this district, and Sakalava is the primarily spoken language. Study participants were from Antsakoamanondro and Antranokarany communities (Fig 1). The villages are situated between 7.5 km and 38 km away from the district capital Ambanja. Since 2014, Madagascar participates in the WHO program on preventive mass chemotherapy with Praziquantel in school-aged children. Since 2015, primary schools within the study area participate in this program. No other control measures have been implemented in the area.

### 2.3 Research team and reflexivity

The core research team was composed of three female researchers: two Malagasy gynaecologists and a German epidemiologist and global health specialist. The Malagasy investigators were familiar with the setting and have previously carried out studies on FGS [12,32]. The investigator from Germany has worked on women’s health and neglected tropical diseases in rural Madagascar [33].

Four midwives, one local health worker and five professional translators composed the support team of the study. The interviewers, transcribers and translators were born in the area and are familiar with the local culture; they were fluent in the Sakalava language. The midwives performed the semi-structured interviews (SSI) and the focus group discussions (FGD). The midwives and one local health worker transcribed the interviews verbatim. The authors added missing words in round brackets, explanatory comments were added in squared brackets. Two of the interviewers and five professional translators translated the interviews from Sakalava into French. Transcriptions and translations were checked for congruency by BSR and OTOR. BSR and OTOR trained the entire support team in qualitative study methods including carrying out, transcribing, and translating qualitative interviews. AS translated the French transcripts into English for purpose of publication.

### 2.4 Study design and recruitment of participants

The qualitative study was embedded in a randomised controlled trial (RCT) in which two doses of praziquantel were compared (https://clinicaltrials.gov/ct2/show/NCT04115072). FGS was diagnosed based on the presence of characteristic clinical pathology at the cervix and/or in the vagina, using a colposcope for magnification and illumination as recommended by Norseth et al. [32].

All women participating in the clinical trial (n = 116) were invited to the qualitative study. Each woman was approached by a community health worker of their villages, and was invited to participate in the study. Seventy-six women accepted to participate and provided written informed consent. According to their preference, women could choose to participate in an individual semi-structured interview (SSI) or a focus group discussion (FGD). Two different methods were offered to give participants the choice between a private or a group setting to talk about their intimate and private experiences with FGS [34].

The authors developed the interview guide, participatory revision was done with the interviewers. The interviewers were not involved in conducting the RCT. The research team chose not to conduct the interviews and FGD themselves to reduce barriers and to avoid social desirability bias and hierarchies.

The theoretical framework of our analysis was based on the “KAP Theory”, a health behaviour change theory based on the interrelated processes of knowledge acquisition, the generation of attitudes, processing of experiences and the formation of behaviour [35,36]. Methodological orientation was the qualitative content analysis of Mayring [37]. The research question guiding the development of our interview guide was *“What do women with FGS in Madagascar know about the disease and what are their experiences related to FGS*?*”*

### 2.5 Data collection

The interviewers carried out SSI and FGD in the villages Antsakoamanondro, Anjavimilay and Ankazokony located in the Ambanja district. The interviews took place in a room of the school or in a communal house in which privacy was guaranteed. No other persons were allowed to attend the interview. Interviewers captured non-verbal communication through written notes in a memo booklet. SSI and FGD were audio recorded. We did not video record to avoid intimidation. Sociodemographic information was retrieved from the corresponding information from the RCT database. The authors revised the interview guide based on the experiences with the first five SSI and the first FGD. SSI and FGD were carried out between the 10th and the 17^th^ of April 2020 in a window of opportunity when COVID incidence in the region was low. Due to time constraints in the context of COVID-19, we carried out in depth data analysis only when all interviews where completed. Therefore, saturation of the data could not be checked. We avoided respondent validation (member checking) of the transcripts to prevent social desirability bias [38].

### 2.6 Data analysis

**Fig 2:**
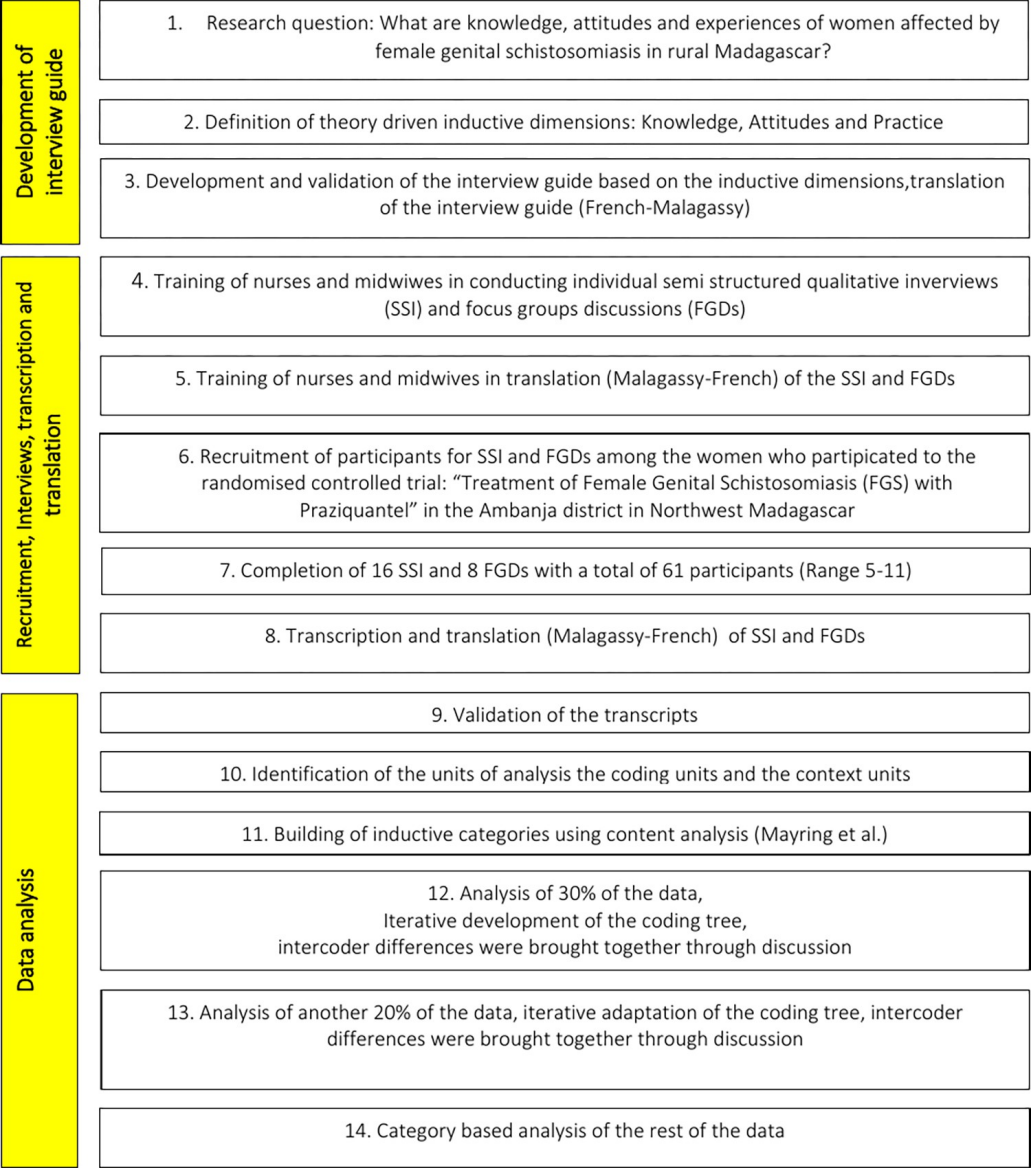
Flow chart of the methodological approach.

After transcription and translation, two authors validated the translated transcripts by comparing them with the audio recordings. There was no systematic difference between FGD and SSI thus both data sources were analysed jointly. Rules to define coding and context units were developed based on the qualitative content analysis [37]: inductive categories were built thematically by paraphrasing and generalising coding units, coding units were then attributed to the deductive categories knowledge, attitudes and practice. Then the text material was reduced in a two-step process into main and secondary categories. The coding tree was built in an iterative manner through the analysis of 20% of the material (3 SSI and 4 FGD). Intercoder differences were brought together through discussions. The iterative adaptation of the coding tree was finalised after the analysis of another 15% of the material (2 SSI and 3 FGD, Fig 2). Two authors carried out category-based analysis of the remaining interviews. Qualitative data were analysed with Microsoft Excel (2010) using the methodological approach in Fig 2. Methods and analysis were performed based on the COREQ recommendations for standardised reporting of qualitative research [39]. Statistical analysis of the sociodemographic data was performed using SPSS (Version 16.0; SPSS Inc, Chicago, Illinois) since data did not follow normal distribution median and range were calculated.

## 3. Results

Of 116 women who participated in the RCT, 76 consented to participate in the qualitative study (66%). Median age was 26.5 years in the SSI and 27.0 years in the FGS group (range 16–35 and 15–35, respectively). The median age among those women with FGS who did not participate was 22 (range 16–35).

**Fig 3:**
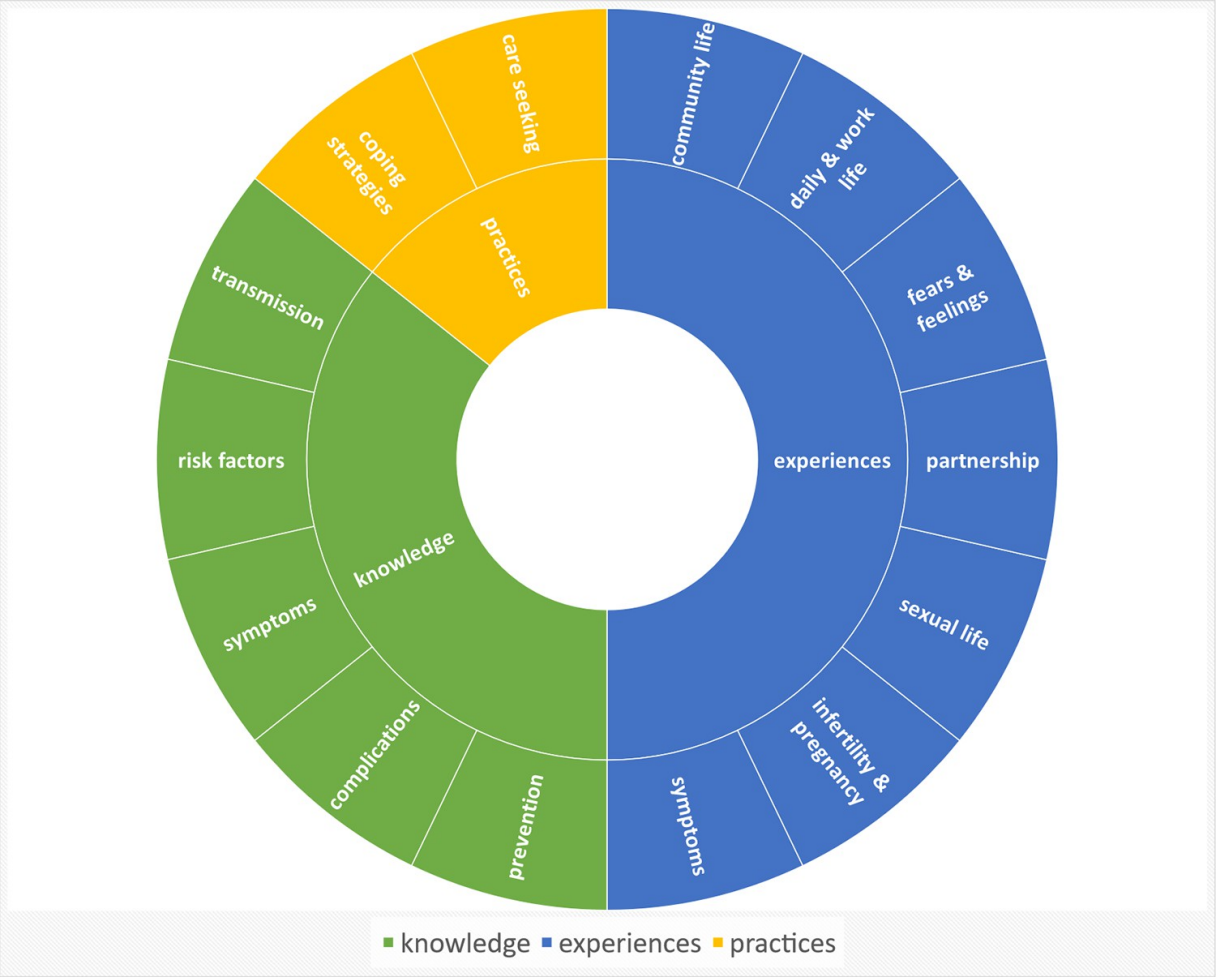
Inductive and deductive main categories and subcategories.

**Fig 4:**
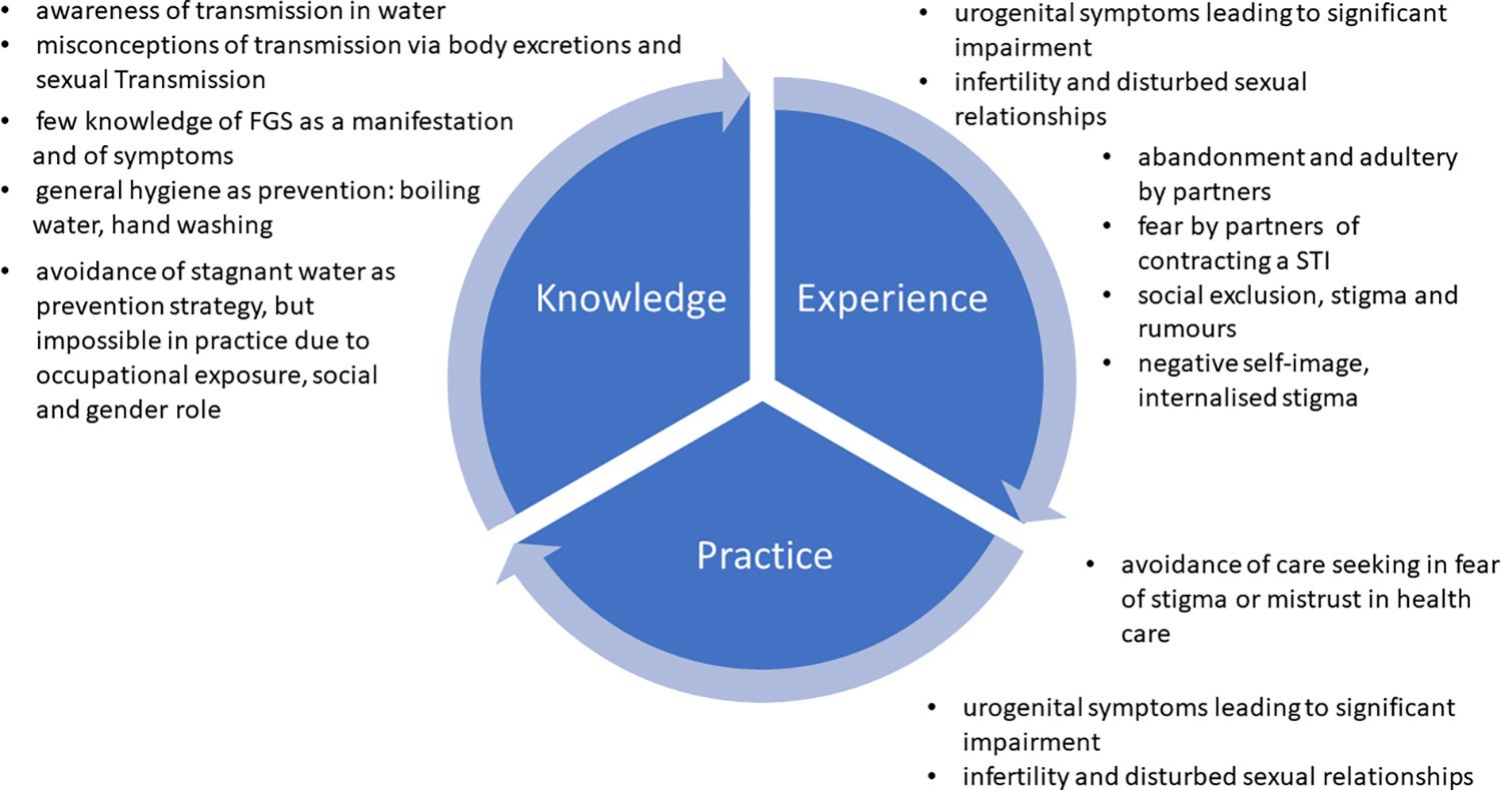
Key results on knowledge experience and practice among women affected by FGS.

Sixteen women took part in individual SSI, 60 women in 8 FGD, each of which included a median of 8 participants (range: 5–11). The median duration of a FGD was 67.5 min (range 48–79 min); the median duration of the SSI was 36 min (range 19–81 min). The sociodemographic characteristics of the participants are summarised in Table 1. Inductive and deductive main categories and subcategories are summarised in Fig 3. Key results are summarised in Fig 4.

**Table 1:** Sociodemographic characteristics of the participants.

	SSI (n = 16)^a^	FGD (n = 8)^b^
Number of participants	16	60
Age (median, range)	26.5 [16–35]	27.0 [15–35]
Ever given birth (yes)	11 (68,7%)	53 (88,3%)
Number of children (median, range)	2 (0–5)	2 (0–8)

^a^ SSI = semi-structured interview

^b^ FGD = focus group discussion

### 3.1 General knowledge on urogenital schistosomiasis

#### 3.1.1 Routes of infection

Most commonly, the infectious agent was named *Bibibibin-draha madinika*, literally translated “a bug penetrating without causing pain”. FGS disease was called *bilharzia*, a term commonly used for schistosomiasis worldwide. While some women thought that any part of the body would be suitable for penetration of the *Bibibibin-draha madinika*, others thought that only when the “*bug”* enters the vagina or the urethra, one gets infected.

“What I know:…it is there,…it comes silently; the way it breaks into us,…the way we catch it is unremarked.” (SSI 08)

“All your genitals are in the water… if you go down deep, the tummy is soaked in water… that’s what causes the blood in the urine.” (SSI 05)

Some assumed that infection occurs when faecal material and/or mud comes into contact with the vagina or the urethra.

"You know we women always pee sitting down. I suspect I got mine in this manner, that’s what I think.” (FGD 07, P5)

Many women carried out intravaginal cleansing and believed that dirtiness of the hands could cause infection.

“During the vaginal wash, it came from our fingernails. So, while you are cleaning your vagina, the thing is entering your body (…). They come from the mud, that’s where the “bugs” come from. (FGD 03, P2)

Several women named sexual intercourse a way for getting infected.

“Hmmm uhhh hahaha, maybe women who have had sex with men; yes, since no woman who has not had sex has been infected …. I think it’s very possible that a woman who has sex with a man could get it." (SSI 02)

However, other women thought that “*bilharzia”* cannot be transmitted sexually.

“Great pox we already know, drip [a common word for gonorrhoea in Madagascar] we already know. But if it is *bilharzia*, the entrance door is different.” (SSI 08)

Some women considered a long-time gap between the infection with *Bibibibin-draha madinika* and the development of complaints plausible.

“When we are still small we bathe in the ponds…: and when we become big it’s inside, when we were still small it is already inside…it used to reproduce in our belly until we were finally big, so there are wounds in the belly.” (FGD 01, P11).

Further, some women were aware of two types of *bilharzia* that have different clinical presentations.

“First of all, there are two kinds of *bilharzia*: the one you get from contaminated water, and the other that affects the womb!" (FGD 2, P7)

“It’s certainly not the same, in my opinion. The child with *bilharzia*, the little boy who pees blood, [and the other that affects the womb]." (FGD 2, P7)

There was uncertainty about the existence of male genital schistosomiasis (MGS).

“We know that *bilharzia* exists, but we don’t know if it affects just women. (FGD 04, P4)

#### 3.1.2 Factors which pose women at risk for getting *bilharzia*

Many women mentioned that working in the swampy lowlands and insufficient body hygiene after water contact could be a risk factor for contracting FGS. For most women the contact with “dirty” water and mud in the river, ponds or rice fields engendered infection.

“The people who are still in their strength who go to work in the swamps, that’s where they catch *bilharzia*. People who plant rice or people (…) who don’t wash off the dirty water. I got the disease because I planted rice in the water.” (FGD 08, P2)

“*Bilharzia* comes from dirty water, that’s where *bilharzia* comes from… it’s from working in the swamps, it goes up from the foot and goes directly into the womb, that’s where it lays its eggs and causes *bilharzia*”. (FGD 04, P2)

Some women considered that using the public washroom or outside defecation could put them at risk for *genital bilharzia*.

“…maybe it’s the toilet (..), we all use the same toilet (…). Because I pee in that toilet, I could have had the infection.” (FGD 4, P4)

Other women suggested that previous pregnancies could play a role.

“Women who have already had children are the most vulnerable with regard to the *bilharzia*.” (FGD 03, P1)

### 3.2 Specific knowledge on *genital bilharzia*

#### 3.2.1 Symptoms and signs

When women were asked what they knew about *genital bilharzia* and how it manifests, they often described the disease as being asymptomatic or as presenting with non-specific symptoms.

“Maybe you think you are in good health. I had a medical check-up before, and the doctor found nothing. I had come back to be examined during the treatment [study]. From the outside it looks like there is nothing, that I was clean and healthy. However, the illness in the belly could not be seen with the naked eye.” (FGD 02, P 6)

“If you are sick, if you don’t see a doctor, you won’t know it’s *genital bilharzia*”. (SSI 08)

Other women described precise signs and symptoms such as bloody vaginal discharge, or pain during sexual intercourse as well as urine contaminated with blood.

“…and if a women is not treated over a long period of time, fluid may come out of the woman’s genitals, perhaps with consequences for your health”. (FGD 03, P3)

“Women are in pain when they urinate (…)… and then when they finish urinating, it’s painful at the end, and afterwards,… it hurts either in the lower abdomen, or in the uterus, I don’t know… and when you have intercourse there is pain in the cervix too; it’s painful, that’s how you recognize it.” (FGD 1, P11)

“…when you have sex with your husband, it hurts. That’s it for us women… . You don´t always pee blood, but when you have sex with your husband, it can hurt.” (FGS 02, P6)

#### 3.2.2 Complications of *bilharzia*

Many women thought that internal genital organs could be affected and harm their babies.

“It’s deadly because those bugs bite you and turn into something else… those bugs… . Sometimes they destroy the womb and the genitals.” (SSI 13)

“…it can damage the womb, … it can. I ‘on’t know… if the woman is pregnant, it will also harm her baby. The person may not be able to have a child if she has genital bilharzia.” (SSI 15)

Some women think that untreated *genital bilharzia* could lead to death.

“…if you d’dn’t treat it and neglected it in the end you may get cancer and die from it.” (FGD 01, P11)

“We are able to do our daily work, and to go where we want, but it grows inside, it eats us from inside. The wound still evolves, and it can reach the womb. It kills. Then, you don’t even know the cause of death. And people would say that you died suddenly, but it was a chronic disease.” (SSI 16)

### 3.3 Knowledge and concepts on how bilharzia could be avoided

Many women were aware that working in the rice fields and in the vegetable gardens along the riverbed would put them at risk for infection with *Bibibibin-draha madinika*. At the same time, they recognise that cultivating rice is the sole source of income and that their daily work in the field is deeply embedded in their culture.

“I don’t have a solution to give. I don’t know. If you say we should avoid going to the fields, it’s their job, mine too! Yes, it’s our livelihood. I don’t know… not to work in the fields, although it’s our job? I don’t have anything else to propose.” (SSI 05)

“My source of income is planting rice, and I’ve already been treated for *bilharzia*, and then it was cured… And we discussed that it was because of the ponds that we caught the disease… So, how will I do my planting?” (FGD 07, P5)

However, several women had ideas in mind on how to reduce or prevent skin contact with water.

“We will still plant, but we take precautions and avoid getting dirty in the water. Then, when you come back from there [the work in the paddy field], you just must shower well. I think… you have to be very careful as soon as the water wets the lower belly.” (FGD 07, P4)

“I stopped working in the pond and now I’m just going to farm in the fields. I don’t work there anymore. Yes, since people told me that you could get the disease this way I stopped going there immediately.” (SSI 02)

Some women named general hygiene measures aiming at preventing faecal-oral transmission, which would not prevent *genital bilharzia*.

“We boil water and drink it, even after it has cooled down it doesn’t matter as it is already boiled.” (FGD 04, P6)

“We must wash our hands with soap, the water is probably dirty, even if it comes from the well, even if the water looks clean, we must always use soap, it kills germs.” (SSI 15)

To avoid contact with *dirty water* with the genitals was thought to prevent *bilharzia*.

“One can, perhaps, say this: one should not soak in the pond, when it is very deep, one must wait until it is less deep so that the water cannot reach the genitals. This is what you should do.” (SSI 16)

### 3.4 Experience women had with genital bilharzia

#### 3.4.1 Disease perceptions

Many women described their complaints like menstrual and premenstrual symptoms; others noted changes in their menstrual cycle.

“Something that had never happened to me before. I never felt my energy being low… like this. I didn’t pee blood; I didn’t have anything, but my energy was weird, like the period was coming. That’s how it was for me.” (FGD 07, P4).

“It looked like I would get my period when I didn’t get my period…… That’s how I felt, in two weeks I felt like that. Totally unusual.” (FDG 07, P4)

“My lower belly hurt, then I had my period. … it lasted almost two weeks, but it was only when I got better (…) my period became regular and lasted four days as usual.” (FGD 08, P5)

Other signs and symptoms named were vaginal discharge, pain, itch and a sensation of a foreign body inside the vagina.

“I also recognise it *[genital bilharzia]* by the losses coming out… it itches when it comes out, and there is a lot of it… It comes out by the thing [vagina] there is a great quantity of disease in the belly, and it comes out like this.” (FGD 01, P11)

“It’s painful and it hurts from the inside, and you don’t really know, and it becomes annoying. (…) I never felt such sensation, an unusual sensation, it’s like if my vagina has become larger and it’s awful.” (FDG 07, P4)

“There is a kind of sand inside (the vagina)… like those little grains inside the body… and then the losses…” (SSI 01)

Other women reported a bloody urine, leucocyturia, a painful micturition or a sensation of heat while urinating, signs and symptoms characteristic of urinary schistosomiasis.

“What made me know that I have *bilharzia* is that every time I go to the toilet, I have a lot of pain in my lower belly. Sometimes when I ride a bicycle and pee afterwards, it is as if the urine is not going to come out, there is pain in my lower belly.” (FGD 06, P 2)

“I used to feel pain when peeing. I would pee blood and pus. I used to have these things before.” (SSI 05)

“You feel pain when peeing, and at the end the urine turns yellow. Then there is blood at the end of the urination… there is, there is blood coming out… then there is pain.” (SSI 4)

Other symptoms mentioned were less organ-specific.

“It’s like when we were giving birth… my belly was hurting, my lower belly was very sore. That’s what happened to me… my lower belly hurt.” (SSI 15)

“I was dizzy, I was dizzy when I still hadn’t got the treatment, yet I was dizzy when I had just worked and when I came home, I was exhausted my body was very tired.” (FGD 01, P4)

Many women reported suffering already for a long time.

“I thought I’ve had this disease for four years, or five years now.” (SSI 08)

#### 3.4.2 Experiences with infertility and pregnancy

Many women reported feeling despair and resignation with the idea of remaining childless.

“They said that I don’t have a uterus. That’s what they were saying over there ([in the health centre]). Eh eh, at the hospital we went to, they said: ‘There is no uterus in it, your uterus is destroyed’. They didn’t examine, but they attended me, and they said there was nothing. (…) I was really desperate, I didn’t even know what I was going to do, I didn’t have any hope of having a child.” (SSI 05)

“Like in my case, not having a child, my husband has his own children. But I don’t know if I can get children. But if it doesn’t happen, what can we do about it? (…) Time is wasted, all the time is just wasted, I doesn’t happen yet, you don’t even see it! You have to prepare yourself psychologically, that’s what I do…” (FGD 05, P4)

Women reported frequent miscarriages, complications during pregnancy and delivery.

“Uhm, for me when I was pregnant, I had a miscarriage all the time. I was already in my eighth month for my first child and then in my seventh month for the second.” (FGD 08, P8)

“When I was walking (…) I had pain in my hips, and in my lower belly… then when I was sitting for a while, I heard it coming out, like blood, going… *psshoak…* like this. Like a person who is about to give birth when their water breaks. There was liquid coming out of it [the vagina], clean liquid”. (SSI 15)

Some women feared complications for the foetus.

“What damage has it done to my child? That’s what I was concerned about… When it’s the mother who has it, that’s one thing. But when it’s your child it’s another.” (FGD 02, P1)

#### 3.4.3 Experiences with sexual life

Many women felt pain during or after sexual intercourse. For some women the pain was so intense that they preferred to abstain from having sexual intercourse.

“Every time I have sex, it hurts. He ([the partner]) doesn’t know how to do it. Mhm… if I have sex with someone, if it will be possible, I’m fine with it, but if ([the penis]) it’s long and oversized, I cannot handle it, because when it’s inside it hurts.” (SSI 08)

“It’s painful and hot in the vagina, and it hurts, when he pulls out it’s very painful.” (FGD 06, P1)

“It is unbearable, you see, as if there would be something boring into an open wound, a wound that has been touched. You see it’s as if there’s something he has hit, that’s how it is. I think that every time it ([the wound]) is touched it can end up killing me… and would rather not do it anymore.” (SSI 08)

Many women also report a certain resignation and accept having sex with their husband to satisfy the sexual desire of the partner.

“But men don’t feel that. They do what they have to do and that’s it. We are the ones who feel the pain. I think so because he doesn’t say: ‘ah, but you are in pain’,… I am the only one who is in pain… I used to tolerate the pain and I would tell him to take it easy.” (FGD 02, P2)

Women who refrained from having sexual relation reported that their partners would have sexual relation outside of the partnership. In some cases, extra marital affairs were outspoken, in other situations they were hidden.

“He was probably angry…eh…., he had gone to look elsewhere. He was asking my permission. He said: ‘I’m going to go there’… and I said: ‘Go ahead’. Yes, because I was ill. And even if I held him back, I couldn’t give him what he wanted…” (FGD 03, P2)

“I cannot trust him, maybe he is seeing another partner somewhere. It is clear he won’t stay with me only. If you don’t see him or you don’t surprise him, he will always deny. (FGD 05, P2)

Some men abstained from having sexual intercourse in fear of acquiring a sexually transmitted disease, other men suspected infidelity of their women.

“Yes, I’ve talked to him about it. We have to go to the hospital, he said…that ([illness]) bothers him… He said: ‘If it was contagious?’ He doesn’t do it [sex] anymore. He doesn’t want it to be transmitted, he told me. He told me to go to the hospital. He works as a driver, he never had time to bring me [to the hospital], so he told me to go.” (SSI 13)

“He said to me: ‘Aren’t you unfaithful?’… no I said… I told him I wasn’t unfaithful.” (SSI 04)

One women suffering from FGS reported that the fear of infecting a partner with FGS has led to withdrawal from dating and sexual relations.

“I even thought it’s a sexually transmitted disease. I don’t trust anymore, every time I go out with someone, suddenly I think that I have an infection… . therefore, I avoid meeting men.” (SSI 08)

“That’s why for a while I did not sleep with anybody. I didn’t sleep with a man for a year.” (SSI 06)

#### 3.4.4 Partnership

Pain during sexual intercourse due to FGS often lead to conflicts with the partner. Some women accepted non-monogamy in their partnership, since they could not fulfil their partners sexual desires.

“I already warned him that ‘my lower belly hurts’. I said, ‘So will you still force me to do it?’ I already had told him that I’m in pain… he did not force me;… ‘Let him see elsewhere, since I’m in pain. I couldn’t sleep with him’ I said.” (FGD 07, P6)

In other cases, *genital bilharzia* led to the separation of the couple.

“We broke up… the reasons for our break-up? I wasn’t feeling well, my lower abdomen hurt. I told him I was sick, and that if he wanted to have sex with me my body wouldn’t be able to take it. When he was still there and I was not able to have sex with him (…) he would say ‘If it’s always like that, I’ll go and look [for a woman] somewhere else’." (FGD 08, P5)

“He always goes away, he left me alone, he was unfaithful during that [disease]… so I took advantage of that … and I left him.” (SSI 08)

Infertility led to distress and abandonment by the partner.

"Yes, that’s it! ‘No child, he said, he separates, and then leave’… In the evening we mate, then there is no baby, then he left me." (FGD 05, P2)

#### 3.4.5 Fears and feelings

Many women reported feeling dirty inside, a feeling, which was independent of the sensation of the presence of a foreign body in the vagina at the cervix.

“My womb was dirty.” (SSI 11)

“It was no longer painful (…). Then, there was nothing [no disease], but it [the vagina], was still dirty.” (SSI 05)

Hopelessness and fear of death because of FGS were reported

“Life deteriorates… this is because, even if you still go to the doctor, it [the disease] still is with you. On this, we lose hope, we tell ourselves that we are going to die, since it is not always curable.” (FGD 5, P2)

#### 3.4.6 Daily life and work

Some women perceived the pain as so intense that they were not able to work or walk long distances. Some of them tried to pursue their work in the field, others had to be supported by family members in their households.

“When we were working on the mountains, I was sick… I had to urinate. My hips hurt. I would say to my father, ‘Daddy, my hips hurt’. He told me I was working too hard, ‘You have hot pee [common term for gonorrhoea in Sub Sahara Africa], he said.” (FGD 03, P3)

“When I walk, everything I do makes me tired or even when I work with my hands, or when I walk a lot, it´s really painful, I can’t stand… in the lower belly, aha I need a massage I said. I felt that all my belly hurts, mainly around the navel area.” (SSI 16)

“My family? … It’s me: only me and my child. She [her girl] is looking after me, just does the work and stuff, because I don’t work… I nearly can´t work.” (SSI 08)

Some women reported high costs for health care which led to economic impairment and debts.

“I said to myself, ah, that has been my illness for a long time [laughs]. Every day when I went to the doctor…, injection, hey heh I said all the money I spent, if I had built a house about three meters with it, it [would have been] finished by now.” (SSI 08)

“I had a little debt because I wasn’t working when it was still sore. You see, you don’t have a lot of funding, just a simple activity. I’m used to getting by, that’s why I’m hiding it a little bit [laughs]. I still have debts regarding my child’s school [fees].” (SSI 08)

#### 3.4.7 Reactions in the community

Some women felt stigmatized because of rumours about their infidelity. While some said they would ignore the gossip, others disclosed they were suffering from it.

“It’s by another woman, and then he slept with me and passed it on to me, that’s what the people at home said.” (FGD 01, P9)

“That’s what they said. But I didn’t give a damn! ‘*[It’s]* because of her infidelity, she couldn’t have children. Ah, because of her husband’s abuse, she couldn’t have children’.” (SSI 05)

“…uhm … it would have killed me, … if they said I have it… *[the disease]*.*”* (SSI 12)

In other cases, not having children led to social exclusion.

“As far as the community is concerned, we are obviously a bit excluded. What can you expect from other people’s children! ‘[They] always ask for favours from other people’s children: She’s the one who doesn’t have children but does nothing else than bossing my children around’." (FGD 05, P2)

Some women reported no or little support by their family or community members

“They just heard me complaining about the pain, they saw me going to the hospital, but they didn’t say anything to me.” (SSI 15)

### 3.5 Health seeking and coping strategies

Many women were hesitant about seeking care at the primary health care centre or at a hospital. Some were afraid of medical procedures other feared to disclose their diseases in the community.

“I would go to see a doctor, but nobody should see where I´m going.” (SSI 12)

“Fortunately, at times I was seen with a big belly, people said I was pregnant, people said that it was because of my pregnancy that I went to the doctor.” (FGD 04, P5)

“I didn’t want to go [to the doctor]…my brother told me, ‘You should go, you don’t even know if they could cure you’.” (FGD 01, P3)

“Mmmm, when I was sick, my mother said: ‘Go to the hospital.’ My belly hurt, there was blood coming out… . I was afraid to have an operation.” (FGS 3, P3)

Many women shared their experiences with other affected women and felt relieved through mutual support.

“For us in the countryside, almost most women complained of being ill. When we were talking to each other [we said]: ‘Oh my dear, you have caught this disease? We don´t reject anyone because we all have the same complaints’.” (FGD 02, P2)

“Everyone explains what they feel in their bodies. There is no discrimination or things like that. We were talking together, and then everyone does her business.” (FGD 02, P3)

They [the other women in the community] told me not to believe it, because a woman without a uterus does not exist. That’s what they said: ‘It will happen, calm down, sometimes it takes a long time.’ Maybe it takes a long time to get pregnant, they gave me courage. “(SSI 05)

Many women reported not talking to anybody or denying the disease to avoid stigmatisation.

“I did not complain to them. I handled the situation on my own. And the people I lived with didn’t say anything, they were silent. … I didn´t complain to the people in the village. Because even if you complain, nobody will help you.” (SSI 02)

Other women accepted the situation as it was and gained strength though faith.

“We didn’t ask God to catch this disease. It just came along. Not everyone who is ill has asked God. It is a natural disease. It comes from God.” (SSI 03)

## 4. Discussion

There are a few studies addressing knowledge and awareness of FGS among health workers and community members in Sub-Saharan. Available studies have been conducted in Ghana and Tanzania [25,26,40].

### 4.1 Knowledge

Women generally considered FGS a waterborne disease but did not mention the exact mode of infection. They assumed that contact with “dirty” water could cause FGS. The intermediate vector of FGS, the Bulinus snail, releasing infectious cercariae was not mentioned. However, the women’s risk perception of contact with “dirty water” as the cause of FGS is in line with a study showing that eutrophic and hypereutrophic waters, which at first impression look dirty, are associated with higher concentrations of freshwater snails, irrespective of the snail genus found [41]. Vaginal washing with dirty hands and unclean water was believed to be a cause of FGS. Although not causal for FGS, practices in which the vagina is washed with soap have shown to be associated with an increased risk of contracting STIs or HIV by reducing the natural barrier function of the vagina [42–44].

Only a few women were aware that cercariae have to penetrate the skin. The pathogenic agent was called *Bibibibin-draha madinika*, “a bug that penetrates without causing pain”. Occupational exposure such as fishing or cultivating rice were considered as a risk for the development of FGS. Sexual transmission was considered another pathway for infection. These concepts are in line with knowledge among lay people and health professionals in Tanzania and Ghana [25,26].

Women precisely described the symptoms of FGs but mentioned unspecific signs such as weight loss. Women thought that untreated FGS could lead to infertility, destruction of genital organs, cancer and eventually death. Circumstantial evidence confirms the assumption of the women of the association between FGS and HPV infection eventually leading to cervical cancer [45,46].

The knowledge of the women with FGS was by far more precise as compared with the knowledge of healthy individuals in Ghana or Tanzania [25,26]. Interestingly, women and girls living in an endemic community in Ghana expressed gynaecological complaints which might be in line with FGS, however, never having heard of FGS as a disease, they did not consider schistosomiasis as the cause [25].

FGS is often considered being sexually transmitted by lay people and health workers alike [25,26,40,47]. Interestingly, some of the affected women in our study mentioned STIs as one possible pathway of FGS transmission of *Bibibibin-draha madinika*. The knowledge gaps reflect the lack of education on FGS in national and school-based health prevention programs [48]. In rural Tanzania, knowledge of FGS was acquired at primary schools and led to a reduction of exposure to the infective agent. Structural measures such as school attendance protected children from acquiring the disease by offering health education and preventing enrolment into domestic duties which might pose them at risk for infection [49].

According to Grant and Shoham [27], education on sexual and reproductive health in Madagascar is shaped by cultural and social rules enforced by the important role of Christian norms. This leads to reduced attainment of girl’s education and gender empowerment. Further, the lack of NGOs with focus on gender has led to limited engagement and investment to improve women’s health [27]. A syndemic approach, which considers interrelated biological and social factors to understand health problems, might be suitable to analyse aggregating dependencies and influences between NTDs, STI and HIV/ AIDS [50]. Thus, not only biological interactions, but also structural and social preconditions shape and influence co-occurrence and risk factors of diseases [50]. With this lens, misconceptions and a lack of health education and stigma constitute a fertile ground for the perpetuation of FGS in disadvantaged communities.

When asked about FGS prevention, the women mentioned measures that were often limited to general hygiene (boiling water before drinking or hand washing) without a specific focus on the transmission of *Bibibibin-draha madinika*. The women named social and economic determinants as the cause of FGS and perceived them as immutable.

Knowledge about symptoms and signs of urinary and genital schistosomiasis was present among the affected women, however, the lack of understanding of the pathomechanisms led to a lack of awareness about prevention in general and primary prevention measures in particular.

Water, sanitation, and hygiene (WASH) related improvements of infrastructure have been proven to be associated with lower FGS prevalence [51]. In our study WASH measures, such as building of latrines were perceived as useful while social and occupational determinants of schistosomiasis were considered immutable. Similarly interesting, a study investigating knowledge, attitude, and practice regarding cutaneous larva migrans in Manaus, Brazil, found that mothers were aware of disease transmission and risk factors such as dirty streets and walking bare foot, but couldn’t translate this knowledge into adequate prevention strategies [52].

### 4.2 Experiences

Many women experienced menstrual-like complaints as well as poly- and dysmenorrhoea. Moreover, vaginal discharge and genital itching were noted. Feeling “dirty inside” and the sensation of the presence of a foreign body in the vagina or at the cervix, reflect characteristic clinical pathology of FGS [32]. Assumably, if grainy sandy patches (which correspond to calcified eggs inside granulomata) cover a large area in the vagina or at the cervix, they could be perceived as “foreign bodies”.

Haematuria and leucocytruia as well as a painful micturition were often mentioned. Pain in the pelvis or the lower abdomen (exacerbated through physical work) had a negative impact on the woman’s working capacity. General symptoms such as fatigue, dizziness and weight loss were often reported and impaired the women’s ability to pursue their occupational duties and household chores. Some women reported having been sick for many years without FGS being considered as the cause of ill health. The reported gynaecological symptoms are in line with those reported in previous studies [8,11,16,47].

Many women in childbearing age experienced infertility, which led to resignation, despair and social exclusion. Some women reported the fear of being rejected by their husbands due to their childlessness. In the Malagasy culture, motherhood is of great social value as it ensures the continuation of kindred and provides evidence of ancestral blessings [31,53]. In contrast to societies in the Global North, where being childless can be an accepted choice or a medical condition which can be addressed, in Madagascar, being a mother is normative to all women and infertility cannot be hidden [54]. Affected women might interpret their infertility as a failure of their body, which might lead to a disturbed self-perception. [55]. However, in a Christian culture such as Madagascar, infertility is not only a negative individual experience. Giving birth is considered an experience common to all women; a social duty [31]. Thus, infertile women are lacking a characteristic which is an inherent part of the women’s community. This leads to exclusion from affective and societal support [56]. The feeling of “incompleteness” is reflected in the multiple expressions in which women with FGS translate their complaints into anatomy and report to be suffering from the “destruction” of internal genital organs.

Pain during sexual intercourse because of FGS was named as a common experience. This symptom was documented previously in clinical studies, [9,11,16,47], however the intensity of impairment and social consequences of the women´s disrupted sexual lives have never been described empirically. Some women accepted to have painful sex to fulfil the sexual desire of the partner, to avoid conflicts or to prevent extra marital affairs or being abandoned. It was common that male partners rejected their spouse, in fear of contracting an STI. Many women expressed resignation and were aware of their partner’s extra marital affairs.

Daily life with FGS affected the women´s capabilities to pursue their daily activities and household chores. In some cases, children took over family duties leading to important imbalances in the family dynamics, as has been shown by children caring for their AIDS sick parents [57].Within the communities, women suffered from social exclusion due to rumours about their unfaithfulness and childlessness. This corresponds to the results of a community study in Tanzania, where women presumably suffering from FGS were referred to as “prostitutes” [26]. Support from family members or community members was rare. Women articulated the three stigma dimensions formulated by Scambler in 2008: the enacted, the anticipated and the internalised stigma [58]. Enacted stigma is displayed by the openly formulated experiences of exclusion in the community, while normalisation of social exclusion, expressed by the perception of childlessness as a logical consequence of social exclusion it to be considered as an expression of anticipated stigma [58]. In contrast to FGS, disclosure to the partner and community member among HIV affected women is increasing, indicating the presence of a social support net and increasing normalisation of HIV diagnosis [59].

Many women affected by FGS expressed feeling “dirty”. Further, the term “dirtiness” was also used to describe the environment in which in the women thought to contract FGS. These findings indicate an internalised form of stigma of the self which is well known in the context of HIV and other STIs [60,61]. In our study group, many women that expressed feeling dirty practiced intravaginal cleansing, which has been shown to be associated with an internalised and enacted stigma among female entertainment workers in Cambodia [62]. Patients with internalised stigma tend to underutilise prevention services and show impaired mental health [58].

### 4.3 Practice

Some women felt so much pressure and rejection that visiting a doctor for FGS screening or treatment seemed unbearable. Comparably, stigmatisation at health care facilities constituted a significant barrier for girls and women seeking health care for reproductive diseases in Ghana [25]. For STIs, rudimentary knowledge and stigma perceived as intense were associated with delayed care seeking behaviour [63]. These barriers could not be addressed adequately through health education and counselling programs [63], indicating that knowledge based interventions might underestimate building of trust into health care providers and the own community [64,65].

Coping strategies, such as seeking peer support or addressing conflicts with the partner, were comparable to those of women with self-reported infertility living in open polygynous marriages in The Gambia [66]. Other, less functional, coping strategies of participants in our study included social withdrawal and denial of their disease. Women also reported separation of the couple due to FGS as well as being single (avoidance of dating and sex).

When women talked about their complaints with others, they felt safe and could cope better with FGS. These results are in line with the findings of Kumar et al. [67] in India, where social support, participation in support networks and selective disclosure are central elements of coping with HIV related stigma. The faith in God was also considered as a source of relief.

According to Sutherst (cited in Bruun and Aagaard- Hansen) sustainable control of vector borne diseases requires, among other measures, community ownership of control measures [68]. However, our study shows that women with FGS do not actively apply prevention strategies. The prevention strategies mentioned are considered effective but cannot be implemented by the women alone and require political and community allies. These findings confirm the structural disadvantages and economic and social vulnerability of women in their community, which have been described in HIV/AIDS and malaria [69,70]. In a WHO report on equity and social determinants of NTDs, the recommended actions include "reducing inequity due to sociocultural factors and gender”[23]. Stigma and isolation are recognized as additional burden that lead to lower acceptance of health services and need to be considered in prevention strategies.

### 4.4 Strengths and weaknesses of the study

Data in this qualitative study were collected from women suffering from FGS who were diagnosed through colposcopy by experienced gynaecologists and who received adequate treatment. Urinary schistosomiasis was present simultaneously in some of the participants of the clinical trial, from which the participants of this qualitative study were recruited. The interviews and focus group discussion were carried out in three villages close to where the women lived. Further willingness to participate might be dependent on personal experiences during the clinical trial and individual responses might be influenced by social desirability. Surprisingly, women reported their sexuality and the socially frustrating experiences related to FGS in clear words. This was probably facilitated by the high level of confidence enabled by local interviewers and the confidential environment during FGDs and SSIs.

Due to dynamics of the Corona virus pandemic in spring 2020, the interviews and FGD were performed in a short time span and could not be analysed immediately after the interviews. Therefore, theoretical saturation was not taken into consideration during data collection. However, due to the large amount of primary data collected, saturation was effectively achieved and no significant new information was acquired in the last interviews.

Nurses and midwives who spoke Sakalava and were familiar with the local culture performed the interviews and moderated the FGDs. However, this might have influenced the validity and information content of the interviews since the interviewers had no previous experiences with this method and the interviews had to be translated from Sakalava to French and then to English.

## 5. Conclusions

The aim of this study was to explore the women’s experiences with FGS and its social consequences in an endemic setting in rural Madagascar.

Women affected by FGS in our study suffered from significant impairment of genital and urinary functions. Massive clinical and social impairment affecting women’s sexual life, partnership, personal communities, and the capability to work and earn their daily living were reported.

Stigma resulted in negative self-image (internalised stigma), and social exclusion within the couple and the community.

Most women were not aware of the pathomechanism and the mode of infection of FGS. Although structural aspects, environmental and social determinants of FGS were mentioned, most women did not see a solution to reduce or eliminate re-exposure.

Our study is perhaps the first to give a voice to women who were diagnosed with FGS and who lived in communities where urogenital schistosomiasis was endemic. It confirms, in a differentiated manner, the spectrum of clinical findings as well as the social consequences of FGS. Our findings highlight the importance of improving access to health education and interventions aiming at improving living conditions, including the supply of water, sanitation and hygiene (WASH). However, beyond educational efforts, studies and interventions aiming at understanding and enhancing trust building in the community and towards the health system are urgently needed. Improved access to diagnosis chemoprophylaxis and chemotherapy with Praziquantel should be provided embedded in adapted prevention, diagnosis and treatment protocols for HIV, HVP and STIs thereby reducing siloed approaches to sexual and reproductive health.
